# Audiologist-Guided Multimodal AI for Pure-Tone Audiometry and Tympanometry Interpretation and Reporting

**DOI:** 10.1007/s10916-026-02463-5

**Published:** 2026-10-01

**Authors:** Xia Wu, Xiaoli Shen, Changgeng Mo, Shengyan Shao, Jie Wang, Shangqiguo Wang

**Affiliations:** 1 Department of Otolaryngology-Head and Neck Surgery, Ningbo Hospital of Integrated Traditional Chinese and Western Medicine, Ningbo, China; 2 School of TCM and Pharmacology Health and Early Childhood Care, Ningbo College of Health Sciences, Ningbo, China; 3 Orka Health Limited, Hong Kong, Hong Kong SAR China; 4 Department of Special Education and Counselling, The Education University of Hong Kong, Hong Kong, Hong Kong SAR China; 5 Department of Otorhinolaryngology, Ningbo Yinzhou District No.3 Hospital, Ningbo, China; 6 Department of Otolaryngology, The Third People’s Hospital of Zhengzhou, Zhengzhou, China; 7 Faculty of Education, The University of Hong Kong, Hong Kong, Hong Kong SAR China

**Keywords:** Artificial intelligence, Clinical decision support, Large language models, Pure-tone audiometry, Tympanometry, Patient communication

## Abstract

Multimodal artificial intelligence (AI) could support audiology reporting, but an end-to-end score can obscure whether errors arise from image transcription, specialty-rule execution, or communication. We evaluated these as separate modules in 155 de-identified outpatient records representing 151 patients. In Module 1, schema-only and audiologist-skill prompts were compared in 50 development records; the locked skill was then tested with a Codex GPT5.5 agentic workflow in 101 independent patients. A post hoc robustness analysis tested matched schema-only and skill-assisted direct-API conditions with DeepSeek-V4-Flash-Vision-Exp in the same 101 patients. The skill increased hearing-loss-type agreement from 85.0% to 94.0% and acoustic-reflex agreement from 70.4% to 98.4% in development. Codex validation yielded 1809/1820 (99.4%) exact numeric thresholds, but only 9/15 (60.0%) no-response entries were correct. In the DeepSeek robustness analysis, the skill increased type agreement from 37.6% to 70.3%, while exact numeric-threshold agreement remained 72.1% and no-response agreement remained 0%. In Module 2, AI workflows received adjudicated structured values, not Module 1 outputs. After two audiologists refined reports using 60 records, 91 independent patients were evaluated. All 25 rule-derived fields were correct in 91/91 Codex and 87/91 DeepSeek cases; both audiologists rated all Chinese reports accurate or basically accurate. These were different operational workflows, not a matched foundation-model comparison. The findings support prospective evaluation of modular, auditable clinician assistance, not autonomous diagnosis, end-to-end performance, or patient usability.

**Supplementary Information:**

The online version contains supplementary material available at https://doi.org/10.1007/s10916-026-02463-5.

## Introduction

Pure-tone audiometry (PTA) and tympanometry underpin hearing-loss and middle-ear assessment. Interpretation integrates frequency-specific air- and bone-conduction thresholds, masking, no response, air-bone gaps (ABGs), tympanogram curves and measurements, acoustic-reflex screening, history, and otoscopy. The same evidence must then be communicated differently to clinicians and patients. Hearing-health materials often exceed recommended reading levels [1], and limited health literacy can coexist with hearing loss in older adults [2]. LLM-assisted rewriting may improve readability [3], clinician-developed plain-language material offers one communication model [4], and further LLM evaluation reinforces the need to preserve clinical meaning [5].

AI research in audiology spans automated assessment [6, 7], professional-knowledge LLM tasks [8, 9], and chatbot performance sensitive to model recency and prompting [10]. Chinese audiologists viewed AI as an auxiliary tool: routine work could be accelerated, but communication, judgment, and oversight should remain clinician-led [11]. Repeatability and evaluator-dependent performance have been studied [12, 13]. Purpose-built systems address audiogram classification [14] and digitization [15, 16], while a complementary classification system illustrates the downstream categorization task [17]. Population analyses also showed that better-ear PTA4 can miss high-frequency and asymmetric patterns, supporting full-frequency review rather than reliance on one average [18]. Separate work predicts middle-ear disease from tympanometric data [19, 20]. Recent multimodal LLM studies reported incomplete audiogram severity and type accuracy and did not support autonomous diagnosis [21, 22]. AUDIOLOGYBENCH likewise found that audiometric numerical interpretation lagged recommendation generation and that task type explained more variation than model selection [23]. A plausible answer can conflate visual extraction, arithmetic, specialty conventions, clinical synthesis, and communication.

Thresholds can be checked cell by cell, derived categories against explicit rules, and narrative reports by human judgment. Separation permits task-specific reference standards and exposes errors hidden by an end-to-end score. Prompt development also varies performance, yet 136 of 137 health-advice chatbot studies omitted a prompt-engineering phase [24]. Medical-imaging AI guidance calls for transparent data provenance, index-test specification, and error analysis [25, 26]. Clinical and chatbot guidance also emphasizes human oversight and reproducible prompt and evaluation details [27, 28].

We therefore developed and internally evaluated two separate modules: (1) schema-only versus audiologist-guided transcription and interpretation of PTA and tympanometry images, followed by a locked test in independent patients and a post hoc cross-model robustness analysis; and (2) rule conformance and Chinese professional and patient-facing reporting from adjudicated structured inputs. We asked where explicit audiology guidance added value, whether its effects transferred across visual-model implementations, and which residual errors limited clinical readiness.

## Methods

### Study Design, Cohort, Ethics, and Governance

**Fig. 1:**
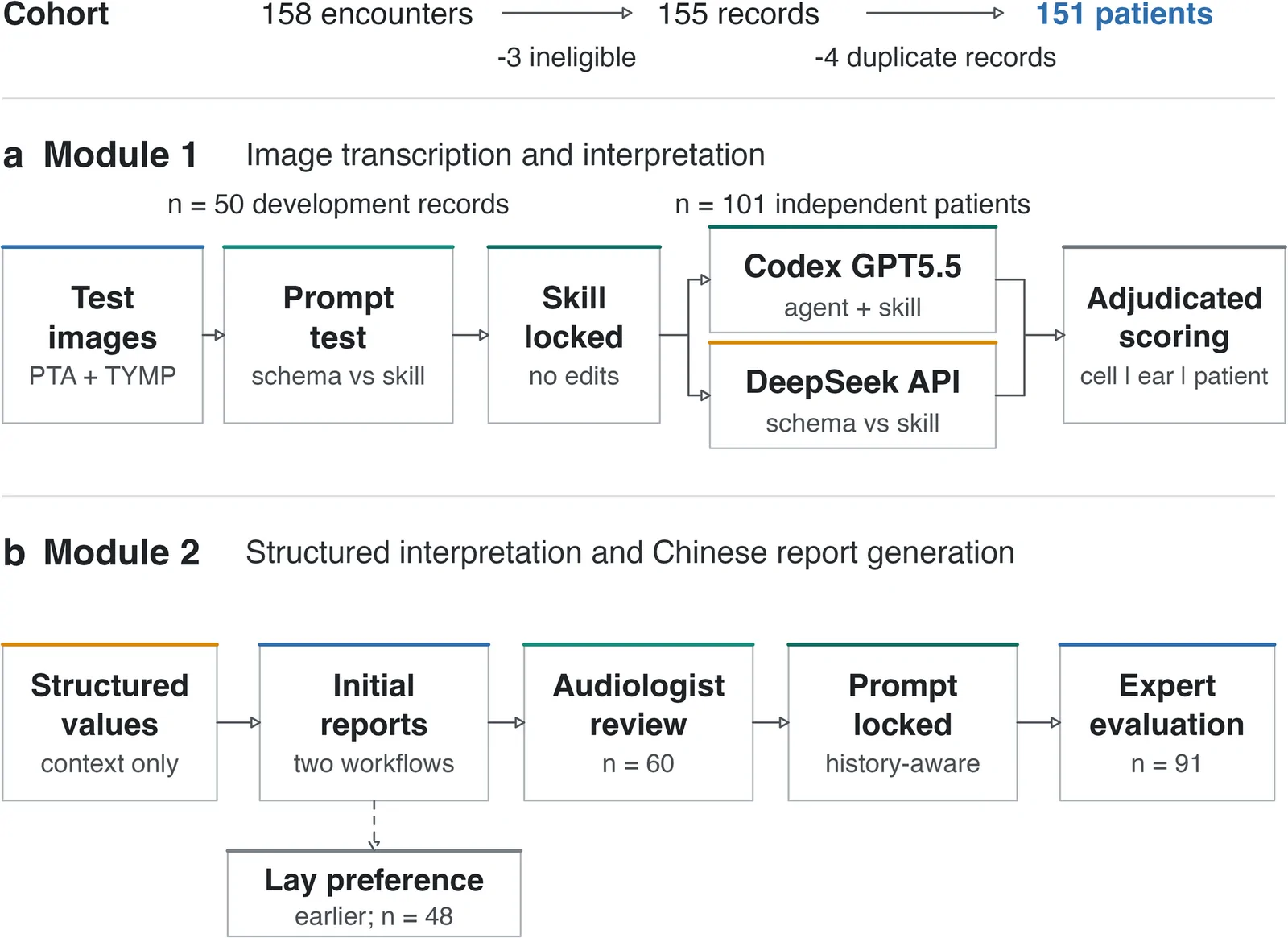
Study workflow, cohort allocation, and component-level evaluation. The cohort line shows derivation from 158 encounters to 155 eligible records representing 151 unique patients. Panel a shows Module 1: 50 development records compared schema-only and skill-assisted prompting before the standard skill was locked. The compact split-and-rejoin branch contains the locked Codex agentic evaluation in 101 independent patients and the post hoc matched DeepSeek schema-only versus skill-assisted API analysis in the same patients; both were scored against the adjudicated reference. Panel b shows Module 2: reporting workflows received adjudicated structured inputs rather than Module 1 predictions; 60 records informed refinement, 91 independent patients entered formal expert evaluation, and the dashed offshoot identifies the earlier pre-refinement lay comparison in 48 patients. The modules were not connected in an end-to-end test

This retrospective component-level development study used 158 outpatient audiology encounters collected from December 2024 through March 2025 at Ningbo Hospital of Integrated Traditional Chinese and Western Medicine, China. Eligibility required bilateral air-conduction PTA3 and professional tympanogram classifications. Three encounters were ineligible, leaving 155 records. A secure identifier audit found four patients with two encounters; retaining the earliest eligible encounter yielded 151 independent patients (302 ears). Patient independence was separately enforced in each final analysis (Fig. 1; Table 1). All eligible records were used without a hypothesis-based sample-size calculation.

**Table 1:** Study modules, inputs, AI conditions, reference standards, and analytic samples

Study phase	Purpose and input	AI condition(s)	Reference/evaluation	Analytic sample
Module 1 development	Separate PTA and tympanometry images	Codex schema only; Codex plus audiologist skill	Adjudicated cell-, ear-, and patient-level reference	50 records
Module 1 locked held-out test	Same image inputs	Codex GPT5.5 agentic workflow plus locked skill	Same adjudicated reference	101 independent patients
Module 1 post hoc cross-model robustness	Same image inputs and response schema	DeepSeek vision direct API, schema only; same API plus locked skill	Same adjudicated reference	101 independent patients
Module 2 refinement	Structured adjudicated values and de-identified context; no images	Codex and DeepSeek reporting workflows	Formative feedback from two audiologists	60 records
Module 2 formal evaluation	Same structured inputs; no Module 1 outputs	Codex GPT5.5 agentic workflow; DeepSeek v4 Flash direct API	Rule conformance and ratings by the same two audiologists	91 independent patients
Module 2 exploratory preference	Pre-refinement patient-facing reports	Same two reporting workflows	Five lay raters and two audiologists	48 independent patients

*PTA* pure-tone audiometry. The two Module 2 conditions differed in both foundation model and execution architecture and were analyzed as implemented workflows, not as a matched model comparison

Direct identifiers were used only for the secure duplicate audit and were removed before access by human research evaluators or AI systems. De-identified context could include age, sex, chief complaint, history, and physical or otoscopic findings; clinic diagnoses were withheld. Ethics approval was obtained from the Human Research Ethics Committee of the University of Hong Kong (EA260116) and the Ethics Committee of Ningbo Hospital of Integrated Traditional Chinese and Western Medicine (YJ260720192039). Individual patient consent was waived for retrospective secondary use under hospital governance. Expert and lay raters gave written informed consent.

### Data Preparation and Reference Standards

PTA used an Otometrics Madsen Astera² audiometer and tympanometry a Madsen Zodiac. Separate de-identified files were rendered at 300 dpi; fixed crops retained axes, legends, symbols, measurements, and reflex fields. In the independent-patient Module 1 set, eight cases required the nearest historical separate file. The two PTA files were each 7 days before the index encounter; six tympanometry files were 6 days, 8 days, approximately 1.5 months, 3 months, 3 months, and 1.5 years earlier (combined median approximately 26.5 days; range, 6 days to approximately 18 months). Sensitivity analyses excluded fallback cases.

Two trained transcribers independently entered air- and bone-conduction thresholds, N/NR, tympanometry measurements, printed curve types, and acoustic-reflex findings while masked to each other and clinic labels. Disagreements were adjudicated from the clearest source. Two audiologists with 13 and 16 years of clinical experience independently classified curves; they agreed in 296/300 dually classified ears (98.7%; Cohen kappa, 0.979), and jointly adjudicated four disagreements and two singly classified ears. Printed and professional curve types remained distinct (Online Resource 1, Table S9).

Thresholds followed the 5-dB HL convention [29]. PTA3 averaged 500, 1000, and 2000 Hz; PTA4 added 4000 Hz. A comparable ABG pair used air- and bone-conduction values from the same ear and nominal frequency with unambiguous ear and masking assignment. Measured responses were used directly; clear N/NR could contribute only through the prespecified maximum-output-plus-5-dB lower-bound rule. Blank, crossed-out or superseded marks, and uncertain symbols were excluded. The prespecified study/local rule defined a meaningful ABG as at least two comparable frequencies with either two gaps > = 15 dB or a mean across all comparable frequencies > = 15 dB; it was informed by clinical convention rather than copied from an external guideline.

Professional A/As/Ad/B/C classification was an expert-consensus, non-rule-based reference standard. The audiologists integrated curve morphology, peak location, static admittance/compliance, and ear-canal volume [30]. Type A had a distinct peak near atmospheric pressure; As and Ad retained a peak but showed relatively shallow and hypercompliant patterns, respectively; B lacked a distinct peak and was interpreted with ear-canal volume; and C had a distinct negative-pressure peak. No universal numerical cutoffs were imposed. Borderline or discordant patterns were jointly reviewed to reach consensus, and the adjudicated research label did not imply that clinical retesting was unnecessary.

Air-conduction PTA3 categories were < = 25 dB HL, 26–40, 41–55, 56–70, 71–90, and > 90 for normal/near-normal through profound loss. Conductive, mixed, and sensorineural categories combined air-conduction PTA3, bone-conduction PTA3, and ABG; insufficient bone data produced indeterminate. In clinic files, N or NR both meant no response at the frequency-specific maximum output; neither meant not tested. Cell-level N/NR remained nonnumeric; only lower-bound PTA3/PTA4, degree, ABG, and type derivation used frequency-specific maximum output plus 5 dB. Blank meant missing or not measured. Under the local 95-dB HL reflex screen, an uncancelled 95 was present, N/NR or a cancelled 95 was absent, and blank/unreadable was missing. Full operational rules and output limits are in Online Resource 1, Table S10.

### Module 1: Image Transcription and Interpretation

The audiologist skill was developed by prompt and process engineering, not fine-tuning. Early errors included non-5-dB thresholds, incorrect degree boundaries, conductive-component overcalling, insufficient bone evidence treated as a negative ABG, and cancelled 95-dB reflex marks read as present. Compact, standard, and detailed skills were compared; the standard version retained all edge rules with fewer tokens (Online Resource 1, Table S1). It required a fixed sequence: verify image and ear, inspect axes and legend, assign air/bone and masked/unmasked symbols, transcribe before calculation, preserve N/NR, then derive and cross-check. Masking was ambiguous when symbol, color, overlap, cancellation, or legend information did not permit unambiguous ear, masked/unmasked, and final-threshold assignment. The prompt then excluded that mark from ABG comparison; this was an abstention rule, not a separately trained or validated detector.

Fifty development records compared the same output schema without specialty rules against the standard skill in isolated Codex tasks. Development errors prompted final clarifications on bone-conduction sufficiency, conservative classification with incomplete bone data, and 500-Hz reflex calls; the skill was then locked. On July 7, 2026, each of 105 residual records underwent one isolated Codex GPT5.5 agent inference. After prespecified deduplication, 101 patients formed the held-out test set. Session logs confirmed access only to the corresponding image pair, without reference or scoring files. The exact model snapshot and sampling parameters were unavailable.

On August 30, 2026, the experimental deepseek/deepseek-v4-flash-vision-exp was used for a post hoc cross-model robustness analysis on the same 105 records and prespecified 101-patient subset. Matched direct-API arms used schema only or the locked skill, temperature 0, disabled reasoning, and JSON output. Two skill-arm responses truncated at 6,000 tokens were retried once with only the output ceiling increased to 12,000. This analysis tested skill portability within one API model; Codex remained a descriptive agentic-workflow benchmark.

### Module 2: Structured Interpretation and Reporting

Module 2 did not consume Module 1 outputs. Both workflows received de-identified context and adjudicated thresholds, no-response flags, tympanometry measurements, printed/professional curve types, and reflex entries. They received neither images nor clinic labels nor precomputed PTA, ABG, degree, type, laterality, or cross-check fields. Each calculated 25 prespecified fields and drafted separate Chinese professional and patient-facing reports. Professional reports had to integrate the full-frequency pattern, history, otoscopy, uncertainty, and next steps. Patient reports used short sentences, common words, direct summaries, and no unexplained abbreviations.

The same two audiologists reviewed 60 initial cases and identified overreliance on PTA3/PTA4, omitted low/high-frequency loss, and weak history-result integration. These requirements were added before the prompt was locked. Of 95 remaining records, exclusion of four patients whose paired encounter had informed refinement left 91 independent patients. DeepSeek v4 Flash (deepseek/deepseek-v4-flash, snapshot deepseek-v4-flash-20260423) was evaluated through OpenRouter at temperature 0; Codex GPT5.5 used one isolated xhigh agentic inference per record. One failed inference per workflow was repeated once. Because model, interface, reasoning controls, and sampling information differed, these were two operational workflows, not a foundation-model experiment.

Reports were randomized as A/B and reviewed with source images by the two audiologists who had refined the prompt. Ratings were 1 (accurate, no obvious problem), 2 (basically accurate, minor wording issue), 3 (clear error), or 4 (severe/potentially misleading). An earlier pre-refinement analysis retained 48 independent patients for exploratory preference ratings by the two audiologists and five uncompensated lay raters recruited through posters without audiology training. Lay participants evaluated only the pre-refinement reports; no post-refinement lay evaluation was planned or conducted.

Direct identifiers remained in hospital-controlled source data solely for duplicate-patient verification; all materials submitted to AI services or evaluators were fully de-identified. DeepSeek requests were transmitted through OpenRouter. The provider selected for report generation could not be independently verified, and image-transcription requests were not restricted to zero-retention providers; provider retention or reuse therefore could not be excluded. Codex and DeepSeek were proprietary services subject to model and platform updates. Dataset and workflow details are summarized in the Dataset Datasheet and Model and Workflow Cards in Online Resource 1; prompts, output schemas, available model identifiers, dates, and retry procedures are reported in Tables S2 and S10.

### Outcomes and Statistical Analysis

Module 1 outcomes were numeric thresholds, N/NR, PTA3, degree, type, ABG, printed/professional tympanogram type, tympanometry measurements, reflex cells, and a patient-level endpoint requiring every numeric and N/NR criterion. Missing predictions were incorrect; N/NR required an empty numeric field plus a positive flag. Development differences used 20,000 bootstrap resamples of whole patients and two-sided patient-cluster randomization tests that exchanged all arm labels within each patient together. The post hoc DeepSeek differences used 20,000 patient-cluster bootstrap resamples without multiplicity-adjusted testing. Locked-test intervals used patient-cluster bootstrap for entry- and ear-level endpoints and Wilson methods for patient-level endpoints. Because the 15 validation N/NR entries arose from only three patients, their point estimates were reported descriptively without confidence intervals.

Module 2 numeric PTA agreement allowed +/-0.15 dB for one-decimal rounding; categorical fields required exact rule agreement, and the patient endpoint required all 25 fields. Report endpoints were ratings 1/2 from both audiologists, rating 1 from both, and any rating 3/4. Workflow-specific proportions, Wilson intervals, observed agreement, and unweighted/quadratic-weighted kappa were descriptive; no between-workflow hypothesis test was used because the execution environments were unmatched. Exploratory preferences used exact binomial tests among decisive patients and paired complexity measures used Wilcoxon signed-rank tests. Sensitivity analyses excluded fallback files and patients younger than 18 years. Analyses were version controlled. Reporting was informed by STARD-AI [26] (Online Resource 2).

## Results

### Module 1: Cohort and Image Transcription

The 151-patient cohort had a mean age of 40.9 years (SD, 17.9; range, 6–90); 97 (64.2%) were female and 13 (8.6%) were younger than 18 years. By air-conduction PTA3, 87 (57.6%) had no speech-frequency loss in either ear, 37 (24.5%) had bilateral loss, and 27 (17.9%) had unilateral loss. Thirty-three (21.9%) had a meaningful conductive component and 98 (64.9%) at least one abnormal professional tympanogram (Table 2).

**Table 2:** Characteristics of the 151-patient cohort

Characteristic	Value
Age, mean (SD), years	40.9 (17.9)
Age, median (IQR), years	42 (26–53)
Age younger than 18 years	13 (8.6%)
Female sex	97 (64.2%)
Chief complaint available	149 (98.7%)
No speech-frequency loss in either ear by AC PTA3	87 (57.6%)
Bilateral hearing loss by AC PTA3	37 (24.5%)
Unilateral hearing loss by AC PTA3	27 (17.9%)
Any clinically meaningful conductive component	33 (21.9%)
Any abnormal professional tympanogram	98 (64.9%)

Values are n (%) unless otherwise indicated. *AC* air conduction, *IQR* interquartile range, *PTA3* mean threshold at 500, 1000, and 2000 Hz

**Fig. 2:**
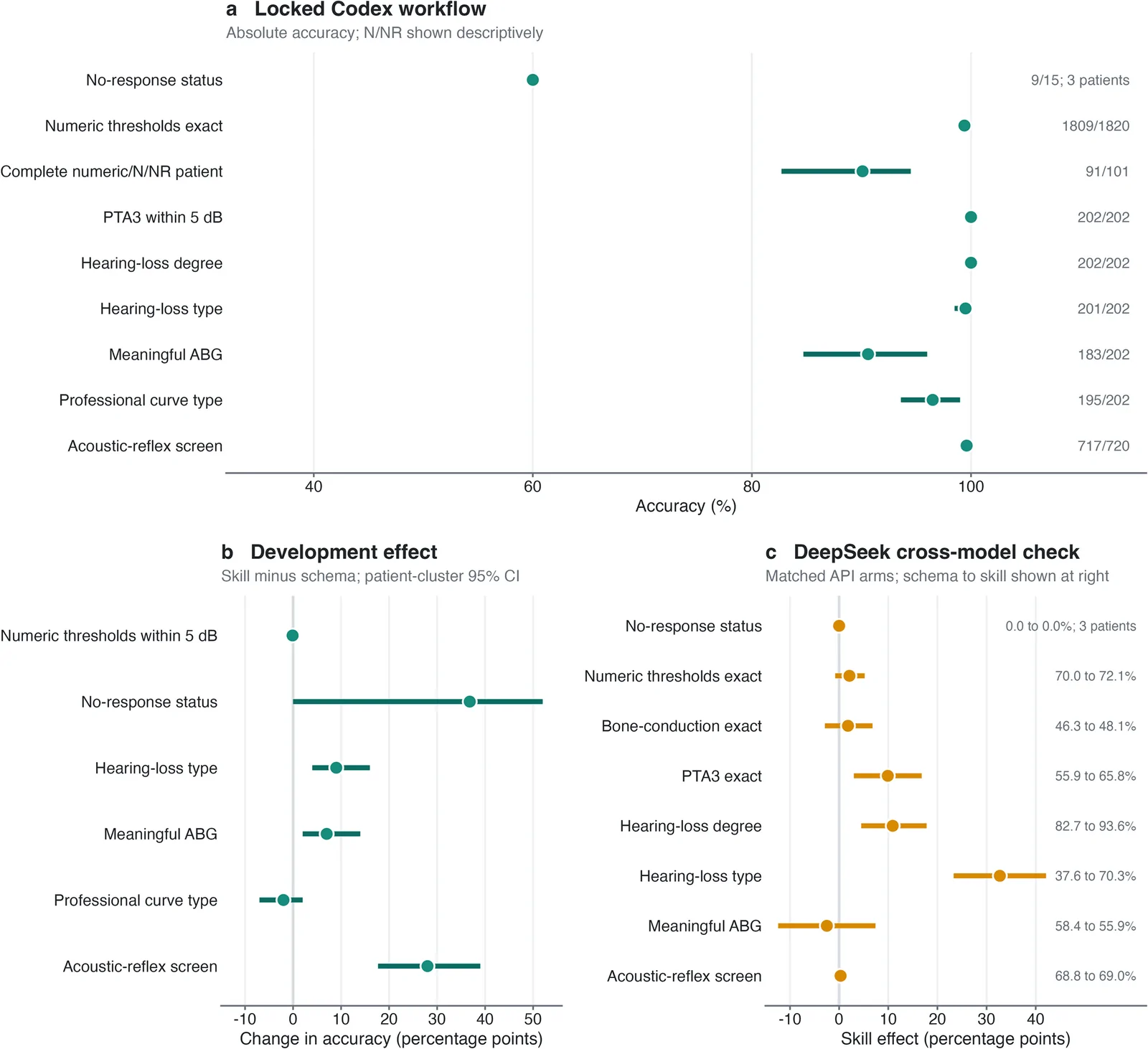
Audiologist-skill image-recognition performance. Panel a shows absolute accuracy for the locked Codex agentic workflow in 101 independent patients. Panel b shows skill-minus-schema accuracy differences in 50 development records with patient-cluster bootstrap 95% confidence intervals. Panel c shows the post hoc matched DeepSeek direct-API comparison in the same 101 patients; positive values favor the skill. Entry- and ear-level intervals use whole-patient bootstrap resampling, whereas patient-level proportions use Wilson intervals. The 15 N/NR entries arose from three patients; these point estimates are descriptive and are shown without confidence intervals. Missing predictions were incorrect, and N/NR required an empty numeric field plus a positive no-response flag. Codex and DeepSeek absolute results are descriptive workflow benchmarks because their execution environments were unmatched. PTA, pure-tone audiometry; PTA3, mean air-conduction threshold at 500, 1000, and 2000 Hz; ABG, air-bone gap; N/NR, no response; pp, percentage points

In 50 development records, numeric-threshold agreement within +/-5 dB was 896/897 (99.9%) with schema only and 895/897 (99.8%) with the skill. Hearing-loss type increased from 85/100 to 94/100 ears (difference, + 9.0% points [pp]; patient-cluster bootstrap 95% CI, + 4.0 to + 16.0; cluster-randomization *p* = 0.0078), and reflex agreement increased from 261/371 to 365/371 cells (+ 28.0 pp; 95% CI, + 17.7 to + 39.0; *p* < 0.0001). ABG increased from 92/100 to 99/100 (+ 7.0 pp; 95% CI, + 2.0 to + 14.0), although the patient-cluster randomization p was 0.0625 because changes occurred in four patients. N/NR increased from 0/19 to 7/19 (+ 36.8 pp; 95% CI, 0.0 to + 52.0; *p* = 0.500), with improvements concentrated in two patients. Professional curve agreement was 94/100 and 92/100 (-2.0 pp; 95% CI, -7.0 to + 2.0; *p* = 0.750) (Fig. 2b; Online Resource 1, Table S2).

In the locked 101-patient Codex test, 1809/1820 numeric thresholds (99.4%; patient-cluster bootstrap 95% CI, 98.9%-99.8%) were exact and 1810/1820 (99.5%) were within +/-5 dB. N/NR was correct in 9/15 entries (60.0%); these entries came from only three patients, so no confidence interval was estimated. Overall, 91/101 patients (90.1%; 95% CI, 82.7%-94.5%) met every numeric/N/NR criterion. Degree was correct in 101/101 patients, type in 100/101, printed tympanogram type in 101/101, and professional curve type in 95/101. ABG was correct in 183/202 ears; 18/19 mismatches were no rather than reference indeterminate when fewer than two comparable bone-conduction frequencies were available. Reflex state was correct in 717/720 cells and tympanometry measurements in 578/578 (Fig. 2a).

The post hoc cross-model DeepSeek analysis gave a different absolute performance level. From schema only to skill, exact numeric-threshold agreement changed from 1274/1820 (70.0%) to 1312/1820 (72.1%; difference, + 2.1 pp; 95% CI, -0.8 to + 5.2), and agreement within +/-5 dB from 87.6% to 90.2% (+ 2.6 pp; 95% CI, + 0.2 to + 5.7). Degree increased from 82.7% to 93.6% (+ 10.9 pp; 95% CI, + 4.5 to + 17.8) and type from 37.6% to 70.3% (+ 32.7 pp; 95% CI, + 23.3 to + 42.1). ABG did not improve (58.4% vs. 55.9%), and N/NR was 0/15 in both arms; no interval was estimated for this three-patient endpoint. Printed type and tympanometry measurements were 100% in both arms; professional curve agreement was 96.0% and reflex agreement approximately 69% (Fig. 2c; Online Resource 1, Table S3).

### Module 2: Structured Interpretation and Reports

With adjudicated structured inputs, all 25 fields were correct in 91/91 Codex-workflow and 87/91 DeepSeek-workflow patients; individual-field agreement was 2275/2275 and 2256/2275, respectively. Both calculated bilateral PTA3/PTA4 exactly. DeepSeek-workflow agreement was 97.3% for ABG, 99.5% for degree, 97.8% for type, 100.0% for tympanogram mapping, and 97.3% for the PTA-tympanometry cross-check; the Codex workflow matched every corresponding rule field (Online Resource 1, Table S4).

**Fig. 3:**
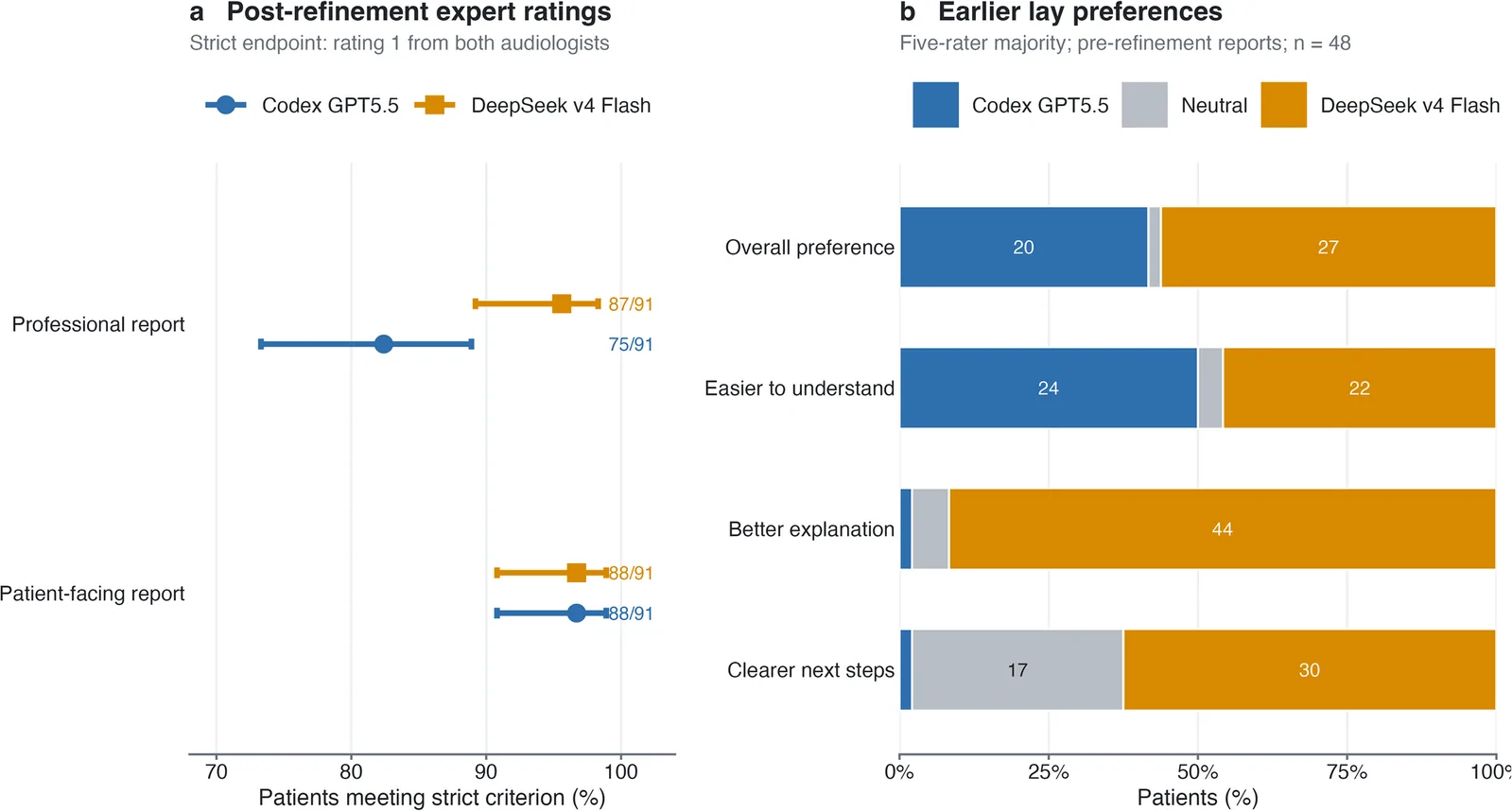
Offline report ratings and exploratory lay preferences. Panel a shows post-refinement strict accuracy, requiring rating 1 from both audiologists, in 91 independent patients; every report from both operational workflows received ratings 1 or 2 from both audiologists and none received rating 3 or 4. Wilson 95% confidence intervals are shown. Panel b shows majority preferences from five lay raters for pre-refinement patient-facing reports in 48 independent patients; ties are neutral. Lay participants evaluated only the pre-refinement reports. Neither panel establishes independent clinical validity, patient comprehension, or prospective safety

Both audiologists assigned ratings 1 or 2 to every professional and patient-facing report from both workflows (91/91 for each; 95% CI, 95.9%-100.0%); no rating of 3 or 4 occurred (Table 3; Fig. 3a). Both assigned rating 1 to 87/91 DeepSeek and 75/91 Codex professional reports, and to 88/91 patient-facing reports under each workflow. These distributions are descriptive because the workflows were not matched. Four-level observed agreement was 0.912–0.967; kappa was unstable where ratings were concentrated near the favorable endpoint. Fallback-file and pediatric sensitivity results were similar (Online Resource 1, Tables S5-S6 and S8).

**Table 3:** Patient-level offline audiologist ratings in 91 post-refinement patients

Operational workflow	Report	Both raters: 1 or 2	Both raters: 1	Any rating 3 or 4
Codex GPT5.5	Professional	91/91 (100.0%)	75/91 (82.4%)	0/91 (0.0%)
DeepSeek v4 Flash	Professional	91/91 (100.0%)	87/91 (95.6%)	0/91 (0.0%)
Codex GPT5.5	Patient-facing	91/91 (100.0%)	88/91 (96.7%)	0/91 (0.0%)
DeepSeek v4 Flash	Patient-facing	91/91 (100.0%)	88/91 (96.7%)	0/91 (0.0%)

A patient had to meet the stated criterion for both audiologists. Confidence intervals and inter-rater measures are provided in Online Resource 1

In the earlier pre-refinement 48-patient package, lay overall preference was 27 DeepSeek, 20 Codex, and one neutral (*p* = 0.382); ease was 22, 24, and two (*p* = 0.883). DeepSeek was preferred for explanation in 44/48 and next steps in 30/48 patients, but its texts were longer: median 392 versus 235 Chinese characters and 28.9 versus 19.4 characters per sentence (both *p* < 0.001) (Fig. 3b; Online Resource 1, Table S7). These findings do not evaluate comprehension of the refined patient reports.

## Discussion

This study treated image transcription and structured-input reporting as separate clinical-AI modules. Audiologist guidance most clearly helped tasks governed by specialty conventions, whereas ordinary threshold reading was already near ceiling in the Codex agentic workflow. The post hoc cross-model DeepSeek analysis showed that the same skill could improve derived categories without reproducing Codex-level transcription. With image uncertainty removed, both reporting workflows followed most rules and produced reports without expert-rated clear errors, but the design did not isolate foundation-model effects or establish patient usability.

### Image Transcription and Atypical Markers

In the patient-clustered development analysis, improvements in hearing-loss type and acoustic-reflex interpretation remained supported when both ears and all cells from a patient were resampled or relabeled together. ABG and N/NR point estimates improved, but patient-cluster randomization was inconclusive because changes were concentrated in few patients. This distinction matters: large numbers of cells do not create equally many independent clinical observations.

The post hoc DeepSeek analysis was a cross-model robustness check, not an independent-dataset validation, because it reused the same cases, reference standard, and skill. The skill improved hearing-loss degree and type, but exact thresholds remained 72.1%, bone-conduction extraction was difficult, reflex agreement was approximately 69%, and N/NR was 0/15. Codex reached 99.4% exact numeric agreement. Because implementations differed, this contrast is descriptive: specialty guidance can improve rule-dependent interpretation while visual accuracy remains environment dependent. Earlier multimodal studies also reported incomplete severity and type accuracy [21, 22], and purpose-built digitization and classification systems separate extraction from downstream categorization [14–17]. Generic chart-to-table and hybrid CV-OCR baselines were not evaluated and remain important future comparators.

Near-ceiling point accuracy was not complete-case reliability. In Codex validation, N/NR agreement was 60.0%, and 18 ABG mismatches occurred because a negative label was forced when the available evidence supported indeterminate. Treating no response as a measured threshold creates false precision; treating absent bone evidence as a negative ABG conceals uncertainty. An interface should expose its cell-level table, distinguish NR from not tested, validate grid and symbol constraints, and abstain on ambiguous masking or insufficient bone data.

Tympanometry marked another boundary. Printed labels and measurements were transcribed accurately, but professional curve classification remained lower and did not improve in development. A printed label is a transcription target; professional typing integrates shape, peak, compliance, and volume. Borderline curves required adjudication even between audiologists and may require retesting clinically. A deployable tool should retain the instrument label, measurements, professional interpretation, and an uncertain/retest option as separate fields.

### Report Generation and Paradigm Constraints

The structured-input module demonstrates rule conformance rather than independent diagnostic validity: reference answers were created from the same prespecified rules supplied to the workflows. Arithmetic and category boundaries could therefore be implemented in unit-tested deterministic code, leaving the LLM to integrate history, express uncertainty, and draft language. Whether that hybrid design is better than either evaluated workflow remains untested.

The refinement feedback also had a substantive audiological implication: a favorable PTA3 or PTA4 category does not make the remaining audiogram dispensable. High-frequency deficits and interaural asymmetry may remain clinically and communicatively relevant when a speech-frequency average appears favorable [18]. A safer reporting architecture should therefore calculate standardized averages but pass the complete frequency-by-ear pattern, relevant symptoms, and otoscopic and middle-ear findings to the narrative layer.

Report findings remain developmental. Both audiologists found every final report at least basically accurate, yet the same audiologists had identified deficiencies and refined the prompt. Their later ratings may therefore reflect shared expectations or incorporation bias and are not an independent clinical validation. DeepSeek and Codex also differed in foundation model, API versus agentic execution, reasoning controls, and available sampling information. Differences in strict rating distributions cannot identify a superior base model and may not persist after model updates.

Lay findings apply only to pre-refinement texts. DeepSeek was preferred for explanations and next steps but produced longer text and sentences; overall preference and ease did not differ. Preference and readability proxies do not establish comprehension. Hearing materials can exceed recommended reading levels [1], and limited health literacy may coexist with hearing loss [2]. Prior hearing-health chatbot studies found completeness, concordance, and readability limitations [31–33]. Although the refined patient reports require shorter, history-linked explanations, their teach-back accuracy, recall, action selection, caregiver comprehension, and accessibility to older or less-educated patients remain unknown.

### Governance, Readiness, and Integration

The modular design makes a future safety architecture visible: image transcription, deterministic validation and calculation, report drafting, uncertainty flags, and clinician approval. It also makes the current boundary explicit. Module 2 received adjudicated values rather than Module 1 output, so no end-to-end error propagation, clinical decision, or patient outcome was tested.

This division is consistent with the role envisioned by Chinese audiologists: AI may absorb repetitive technical work, while clinicians retain contextual judgment, counseling, and responsibility [11]. In practical use, an assistant should present transcribed values, rule outputs, and uncertainty for approval rather than silently collapsing them into a diagnosis. These visible handoff points also permit correction before report generation or patient communication.

Reproducibility was constrained by reliance on proprietary services. Although prompts, output schemas, available model identifiers, dates, and retry rules are reported, the exact Codex snapshot and sampling settings were unavailable, the provider used for report generation could not be independently verified, and image-transcription requests were not restricted to zero-retention providers. These factors may affect reproducibility and governance as services evolve; hearing-health chatbot performance also varies with model recency and prompting [10]. Clinical implementation would require contracted services with explicit retention and data-location terms, access controls and logs, version management where possible, regression testing, uncertainty display, and clinician review.

Other limitations were one hospital, two devices, four months, only 15 N/NR entries in three validation patients, historical fallback files, limited pediatric representation, an enriched nonrandom development set, two involved audiologist raters, and five lay raters. Zero ratings of 3/4 among 91 patients still permits an unfavorable-report rate of approximately 4.1%. Workflow time, correction burden, equity, comprehension, management changes, and outcomes were not measured. Prospective silent deployment should connect the modules while preserving intermediate outputs and measure abstention, corrections, review time, comprehension, and downstream decisions [27, 34]. Its interface should also make uncertainty and intermediate reasoning inspectable rather than presenting an unexplained final answer [35]. External validation should include multiple sites and devices and enrich NR, masking, severe loss, borderline tympanograms, pediatric cases, and incomplete histories.

## Conclusion

Audiologist guidance improved several convention-dependent image tasks, but its effect and absolute accuracy depended on the implementation, and no-response handling remained unreliable. Structured-input workflows showed high rule conformance and favorable developmental expert ratings. Prospective evaluation must now test the connected modules, independent users, patient comprehension, and governed deployment; this study does not establish autonomous diagnosis, end-to-end performance, or clinical effectiveness.

## Supplementary Information

Below is the link to the electronic supplementary material.

Supplementary Material 1

Supplementary Material 2

## Data Availability

Raw clinical records, source test images, and case-level clinical context cannot be shared because hospital data-governance and research-ethics approvals prohibit public disclosure. Access to de-identified derived case-level data would require separate hospital and ethics approval. Aggregate data are reported in the article and Online Resource 1. The non-identifiable audiologist-skill prompts used in the formal evaluations and an integrity manifest with version identifiers and SHA-256 hashes are available in GitHub release v1.0.0 at https://github.com/sqgwang/audiologist-skills/releases/tag/v1.0.0. Analysis scripts can be made available with the non-identifiable prompt materials.

## Declarations

### Generative AI and AI-Assisted Technologies in the Writing Process

OpenAI Codex was used to assist with code generation, language editing, reference-format checks, and manuscript formatting. All analyses, interpretations, citations, and manuscript text were reviewed and approved by the authors, who take full responsibility for the content.

### Trial Registration

Clinical trial number: not applicable.
