# Supplementary material for: Audiologist-Guided Multimodal AI for Pure-Tone Audiometry and Tympanometry Interpretation and Reporting
Source: J Med Syst. 2026 Oct 1;50(1):140. doi: 10.1007/s10916-026-02463-5 (PMC13630873; doi:10.1007/s10916-026-02463-5)
Supplement: Supplementary file 1 [file 10916_2026_2463_MOESM1_ESM.docx]

**Online Resource 2: STARD-AI Reporting Checklist**

Article: Audiologist-Guided Multimodal AI for Pure-Tone Audiometry and Tympanometry Interpretation and Reporting

Journal: Journal of Medical Systems

Authors: Xia Wu^1^, Xiaoli Shen^2^, Changgeng Mo^3,4^, Shengyan Shao^5^, Jie Wang^6^, Shangqiguo Wang^7^

Affiliations: ^1^Ningbo Hospital of Integrated Traditional Chinese and Western Medicine; ^2^Ningbo College of Health Sciences; ^3^Orka Health Limited; ^4^The Education University of Hong Kong; ^5^Ningbo Yinzhou District No.3 Hospital; ^6^The Third People's Hospital of Zhengzhou; ^7^The University of Hong Kong

Corresponding authors: Xiaoli Shen (shenxiaoli0574@outlook.com); Shangqiguo Wang (sqgwang@hku.hk)

The checklist uses the 2025 STARD-AI item numbering. The study is an internal component-level evaluation rather than an autonomous diagnostic deployment; non-applicable and incompletely assessed items are identified explicitly.

| **Item** | **Reporting requirement** | **Location or reporting status** |
| --- | --- | --- |
| 1 | Identify the AI diagnostic-accuracy evaluation and report an accuracy measure | Title/Abstract |
| 2 | Summarize design, methods, results, and conclusions | Title/Abstract |
| 3 | Describe clinical background, intended use, and workflow role | Introduction |
| 4 | State objectives and hypotheses | Introduction |
| 5 | State retrospective or prospective data collection | Methods |
| 6 | Report ethics approval | Methods |
| 7 | Give participant- and data-level eligibility criteria in order | Methods |
| 8 | Explain how potentially eligible participants were identified | Methods |
| 9 | Report setting, location, and dates | Methods |
| 10 | Describe consecutive, random, or convenience sampling | Methods |
| 11 | Describe data source and whether routinely collected | Methods |
| 12 | Describe annotators, expertise, and annotation process | Methods |
| 13 | Report acquisition devices and index-test software/model information | Methods |
| 14 | Describe acquisition and preprocessing for replication | Methods; Online Resource 1 (fixed 300-dpi rendering and crop contents; patient image packets are restricted) |
| 15a | Describe the AI index test sufficiently for replication | Methods; Online Resource 1 (skill, schema, settings, and provenance; Codex snapshot and sampling metadata were unavailable) |
| 15b | Describe development, validation, testing, and sample sizes | Methods |
| 15c | Define and justify cutoffs or result categories | Methods |
| 15d | Specify intended end user and required expertise | Introduction; Discussion |
| 16a | Describe the reference standard sufficiently for replication | Methods |
| 16b | Explain why the reference standard was selected | Methods; Online Resource 1 (expert-consensus reference standard, rationale, and limitations) |
| 16c | Define reference-standard cutoffs and categories | Methods |
| 17a | State what clinical/reference information was available to index-test performers | Methods |
| 17b | State what clinical/index-test information was available to reference assessors | Methods; Online Resource 1 (masking of transcribers and audiologist curve-classification procedures) |
| 18 | Describe accuracy estimation and comparison methods | Methods |
| 19 | Explain handling of indeterminate results | Methods |
| 20 | Explain handling of missing data | Methods |
| 21 | Describe variability analyses and label prespecified versus exploratory | Methods |
| 22 | State intended sample size and determination | Methods |
| 23 | Describe error analysis and any bias/fairness assessment | Methods; Discussion (error analysis reported; fairness was not assessed) |
| 24 | Show participant flow | Fig. 1 |
| 25 | Report demographic, clinical, and technical characteristics by partition | Table 2; Online Resource 1, Table S8 (the 48-patient lay subset was not separately characterized) |
| 26a | Report severity distribution among target-condition cases | Table 1; Online Resource 1 |
| 26b | Report alternative diagnoses among cases without the target condition | Not applicable: component-level audiology categories rather than alternative disease diagnoses |
| 27 | Report time interval/interventions between index and reference tests | Methods; Results |
| 28 | Discuss representativeness of the intended-use population | Methods; Discussion |
| 29 | For external evaluation, compare datasets | Not applicable: internal single-source evaluation; external validation is proposed |
| 30 | Cross-tabulate or distribute index results by reference results | Results; Online Resource 1, Tables S2-S5 (endpoint distributions reported; no disease-level cross-tabulation) |
| 31 | Report accuracy estimates with precision | Methods; Results; Online Resource 1, Tables S2-S5 (N/NR point estimates have no CI because only three patients contributed) |
| 32 | Report adverse events from index/reference testing | Not applicable: retrospective non-interventional evaluation |
| 33 | Discuss bias, uncertainty, and generalizability limitations | Discussion |
| 34 | Discuss intended clinical role and practice implications | Discussion |
| 35 | Address ethics, standards, and fairness | Methods; Discussion (ethics and governance reported; fairness was not assessed) |
| 36 | Report registry name and number | Main manuscript: Trial Registration |
| 37 | State where the full protocol can be accessed | No standalone full protocol was registered; methods are reported in the manuscript and Online Resource 1, and non-identifiable skill materials are linked in Data, Material, and Code Availability |
| 38 | Report funding/support and funder role | Main manuscript: Funding |
| 39 | Report commercial interests | Main manuscript: Competing Interests |
| 40a | State dataset/code availability and reuse restrictions | Main manuscript: Data, Material, and Code Availability; Online Resource 1 |
| 40b | State whether outputs are stored and auditable | Main manuscript: Data, Material, and Code Availability; Online Resource 1 |
