# Supplementary material for: Audiologist-Guided Multimodal AI for Pure-Tone Audiometry and Tympanometry Interpretation and Reporting
Source: J Med Syst. 2026 Oct 1;50(1):140. doi: 10.1007/s10916-026-02463-5 (PMC13630873; doi:10.1007/s10916-026-02463-5)
Supplement: Supplementary file 2 [file 10916_2026_2463_MOESM2_ESM.docx]

**Online Resource 1: Supplementary Methods, Model Cards, Dataset Datasheet, and Results**

**Article:** Audiologist-Guided Multimodal AI for Pure-Tone Audiometry and Tympanometry Interpretation and Reporting

**Journal:** Journal of Medical Systems

**Authors:** Xia Wu^1^, Xiaoli Shen^2^, Changgeng Mo^3,4^, Shengyan Shao^5^, Jie Wang^6^, Shangqiguo Wang^7^

**Affiliations:** ^1^Department of Otolaryngology-Head and Neck Surgery, Ningbo Hospital of Integrated Traditional Chinese and Western Medicine, Ningbo, China; ^2^School of TCM and Pharmacology Health and Early Childhood Care, Ningbo College of Health Sciences, Ningbo, China; ^3^Orka Health Limited, Hong Kong, Hong Kong SAR, China; ^4^Department of Special Education and Counselling, The Education University of Hong Kong, Hong Kong, Hong Kong SAR, China; ^5^Department of Otorhinolaryngology, Ningbo Yinzhou District No.3 Hospital, Ningbo, China; ^6^Department of Otolaryngology, The Third People's Hospital of Zhengzhou, Zhengzhou, China; ^7^Faculty of Education, The University of Hong Kong, Hong Kong, Hong Kong SAR, China

**Corresponding authors:** Xiaoli Shen, shenxiaoli0574@outlook.com; Shangqiguo Wang, sqgwang@hku.hk

This online resource contains the methodological and numerical detail supporting the main article. It is not a patient-level dataset and contains no source clinical images, direct identifiers, or case-level clinical narratives.

# **Supplementary Methods**

## **Additional Cohort Allocation and Fallback Inventory**

The source contained 158 encounters, of which 155 met the record-level eligibility criteria. A secure direct-identifier audit found four patients with two eligible encounters. The earliest encounter from each pair was retained for the 151-patient cohort. Task-specific exclusion of a residual paired encounter yielded 101 patients for the locked and post hoc image analyses, 91 for the post-refinement report evaluation, and 48 for the earlier preference analysis. Development records already used for prompt editing or expert feedback were not retrospectively replaced.

Ethics approval was obtained from the Human Research Ethics Committee of the University of Hong Kong (EA260116) and the Ethics Committee of Ningbo Hospital of Integrated Traditional Chinese and Western Medicine (YJ260720192039). Individual patient consent was waived for retrospective secondary use under hospital governance. Expert and lay raters provided written informed consent.

Eight cases in the 101-patient image set lacked a same-visit separate test file. Two PTA files were each 7 days before the index encounter. Six tympanometry files were 6 days, 8 days, approximately 1.5 months, 3 months, 3 months, and 1.5 years earlier. Across all eight, the median interval was approximately 26.5 days and the range was 6 days to approximately 18 months. Sensitivity analyses excluded these cases.

## **Additional Reference-Standard Procedures**

Two trained transcribers independently entered frequency-specific air- and bone-conduction thresholds, no-response notation, tympanometry measurements, instrument-printed tympanogram type, and acoustic-reflex entries. During first-pass transcription, each was masked to the other's entries and to the clinic diagnostic label. Disagreements were checked against the clearest available separate source file and adjudicated.

Professional A/As/Ad/B/C classification was designated an expert-consensus, non-rule-based reference standard. Two audiologists with 13 and 16 years of clinical experience independently assigned labels by integrating morphology, peak location, static admittance/compliance, and ear-canal volume. No universal numerical cutoffs were imposed. Instrument-printed type and professional type were retained separately because the former is a transcription target and the latter is a clinical judgment. Borderline or discordant curves were jointly reviewed to reach consensus for benchmark construction; this did not imply that clinical retesting would be unnecessary.

Threshold values were constrained to 5-dB HL steps. N/NR remained missing as an observed threshold; only when a lower-bound derived calculation was required did the benchmark use the local maximum output plus 5 dB. Local maximum air-conduction outputs at 250, 500, 1000, 2000, 4000, and 8000 Hz were 105, 120, 120, 115, 120, and 105 dB HL. Bone-conduction maxima at 250, 500, 1000, 2000, and 4000 Hz were 50, 70, 80, 80, and 75 dB HL. These rules were specific to the local test workflow and should not be generalized without device and protocol verification.

## **Audiologist-Skill Development**

The audiologist skill was developed iteratively from observed failure modes; it was not fine-tuning and did not change model weights. Early development testing exposed five recurrent problems: non-5-dB threshold values, left/right or symbol confusion, incorrect hearing-degree boundaries, overcalling a conductive component when bone-conduction evidence was incomplete, and reading a struck-through 95-dB reflex mark as a present response. The skill therefore encoded a read order, graph-grid constraints, conventional air- and bone-conduction symbols, masking and no-response rules, ear-specific checks, explicit missingness, ABG sufficiency criteria, conservative hearing-loss type assignment, tympanometry transcription versus curve judgment, and reflex cancellation rules.

Three variants were prepared. The compact version prioritized the smallest prompt that covered common rules. The standard version included the full read order, cross-checks, uncertainty requirements, and structured output schema. The detailed version added longer explanatory guidance and examples. Rule-coverage auditing and six stress records drawn from a 20-record early-development subset showed that the standard and detailed versions covered the same edge rules; the standard skill was retained because it required fewer tokens.

The 50-record development set comprised 20 prespecified common or difficult patterns and 30 records selected by a deterministic coverage algorithm. The algorithm used only adjudicated reference features to enrich underrepresented hearing degrees and types, ABG states, tympanogram classes, printed-versus-professional discrepancies, laterality, no-response, and rare configurations. It was not intended to estimate prevalence. The residual pool was not used for prompt editing after the skill lock.

Schema-only and standard-skill conditions used the same images, structured response fields, output constraints, and isolated short-context workflow. The schema-only prompt identified required fields and formatting but omitted audiology interpretation rules. The standard skill added the specialty guidance. Development mismatches led to three final clarifications: do not call ABG absent when fewer than two comparable bone-conduction frequencies are available; assign hearing-loss type conservatively around PTA3 values of 26-30 dB HL when bone data are incomplete; and require clear evidence before calling a 500-Hz reflex present. These changes produced the locked skill.

## **Run Isolation and Post Hoc Cross-Model Visual Robustness**

The locked recognition run was executed on July 7, 2026 using one Codex GPT5.5 agent task per record. Each task packet named only the corresponding PTA and tympanometry images and prohibited access to reference files, adjudication data, prior outputs, evaluations, scoring files, and other skills. The response schema required observed threshold and N/NR values, derived PTA3, degree and type, ABG, tympanometry measurements, printed and curve-based types, reflex cells, missingness, and uncertainty.

All 105 record outputs were matched to unique archived sessions. Logged tool traces showed that every session opened both permitted images, none used an unexpected tool, and none accessed a path labeled as reference, adjudication, scoring, or evaluation. Four sessions additionally opened a temporary crop derived from the same permitted record. The workspace was not an operating-system filesystem sandbox, so the audit verifies recorded tool behavior rather than absolute technical inaccessibility. The patient-independent analysis used 101 of these outputs.

The public release contains the exact non-identifiable skill, schema, and integrity hashes. It does not contain patient images, case prompts, or outputs. Codex did not expose a stable public model snapshot or conventional sampling parameters; GPT5.5, the task date, access interface, reasoning setting where available, and observed tool trace were therefore reported without inferring unavailable metadata.

On August 30, 2026, the same 105 packets were processed through OpenRouter with the experimental deepseek/deepseek-v4-flash-vision-exp. Matched direct-API arms used the same schema either without specialty guidance or with the locked skill. Temperature was 0, reasoning was disabled, and JSON output was required. Two skill-arm responses truncated at 6,000 tokens were retried once with only the maximum output increased to 12,000. The same four paired-encounter exclusions yielded the pre-existing 101-patient analysis set. Zero-retention routing was not enforced, so downstream retention or training use could not be excluded.

## **Additional Structured-Input and Report Procedures**

Module 2 intentionally bypassed visual extraction. Each input packet contained frequency-specific adjudicated air- and bone-conduction values and N/NR flags; tympanometric pressure, admittance/compliance, ear-canal volume, instrument-printed type, and professional curve type; acoustic-reflex entries; and de-identified clinical context where available. Precomputed PTA3/PTA4, ABG, degree, type, laterality, asymmetry, tympanometry cross-checks, and clinic diagnoses were withheld.

The required structured response contained 25 fields spanning bilateral PTA3/PTA4, clinically meaningful ABG, hearing-loss degree and type, tympanogram abnormality and mapping, PTA-tympanometry cross-check, worse/better ear, hearing-loss laterality, conductive-component laterality, and middle-ear-evidence laterality. Reference answers were generated from the prespecified benchmark rules. This design measured rule conformance under correct inputs and should not be interpreted as independent diagnostic validation.

The professional report prompt required a concise ear-specific synthesis of full-frequency PTA, degree and type, clinically meaningful ABG, tympanometry, reflex screening, clinical-history alignment, uncertainty, and appropriate next steps. The patient-facing prompt required short Chinese sentences, common words, a direct summary before detail, no unexplained abbreviations, no unnecessary Hz/dB lists, preservation of clinically relevant low- or high-frequency findings even when PTA3 was normal, and advice to confirm decisions with a clinician.

Initial outputs were automatically compared with the 25 rule-derived fields. Two audiologists then reviewed reports from 60 records. Their feedback identified overreliance on PTA3/PTA4, omission of low- and high-frequency deficits, and insufficient integration of symptoms, history, otoscopy, PTA, and tympanometry. The final prompt added these requirements and was locked before the 95-record post-refinement run. The final patient-independent analysis excluded four paired encounters and contained 91 patients.

## **Human Rating and Preference Procedures**

Formal post-refinement outputs were randomized as Version A or B. The two audiologists independently viewed the source PTA and tympanometry images, available de-identified clinical context, and each AI report. They assigned one four-level accuracy rating to the professional report and one to the patient-facing report: 1, accurate with no obvious problem; 2, basically accurate with a minor wording or completeness issue; 3, clear error, usually without serious risk; or 4, severe or potentially misleading. The primary endpoint required both audiologists to assign 1 or 2. The stricter endpoint required both to assign 1. The completed files were checked for missing ratings and locked before patient-level reanalysis.

The exploratory preference package was created before report refinement and therefore represents development-phase outputs. Two audiologists compared professional and patient-facing reports across overall quality, accuracy, clarity or understandability, and next steps. Five lay raters were recruited through open poster advertisements, had no formal audiology education or training, and received no compensation. They compared patient-facing texts for overall preference, ease, explanation, and next-step clarity. Hearing status and health literacy were not collected.

## **Additional Statistical Detail**

Numeric recognition was reported at threshold, ear, and patient levels. Exact agreement and agreement within +/-5 and +/-10 dB were calculated with missing outputs classified as incorrect and coverage reported separately. N/NR was correct only when the numeric field was empty and the no-response flag positive. Development confidence intervals used 20,000 bootstrap resamples of whole patients. Two-sided patient-cluster randomization tests swapped all schema-only and skill labels within each patient together; exact enumeration was used when feasible and otherwise 200,000 Monte Carlo permutations were used. Locked-test entry- and ear-level intervals and post hoc DeepSeek differences used 20,000 patient-cluster bootstrap resamples; patient-level proportions used Wilson intervals. No interval was estimated for validation N/NR performance because the 15 entries arose from only three patients. No multiplicity-adjusted hypothesis tests were performed.

For formal report ratings, patient was the unit of analysis. Workflow-specific proportions, Wilson intervals, observed agreement, and unweighted and quadratic-weighted kappa were descriptive. Because the two workflows differed in model and execution architecture, no between-workflow hypothesis test or effect estimate was used. The complementary upper 95% bound for zero unfavorable reports among 91 patients was approximately 4.1%.

Preference used two-audiologist consensus or five-lay-rater majority. Ties and absence of expert consensus were neutral. Exact two-sided binomial tests among decisive patients were exploratory and not adjusted for multiplicity. Paired text-complexity measures used two-sided Wilcoxon signed-rank tests. Chinese text-complexity proxies used character count, characters per sentence, medical terms per 100 Chinese characters, abbreviations, and numeric tokens. They were not validated education-grade readability measures.

# **Dataset Datasheet**

| **Item** | **Description** |
| --- | --- |
| Intended use | Internal development and component-level evaluation of clinician-assistive PTA and tympanometry interpretation and reporting |
| Source | Routine outpatient audiology records from one hospital in Ningbo, China |
| Collection period | December 2024 through March 2025 |
| Source unit | Audiology encounter; final statistical units were deduplicated patients within each evaluation set |
| Eligible records/patients | 155 eligible records; 151 unique patients overall |
| Modalities | PTA image, tympanometry/reflex image, structured measurements, and limited de-identified clinical context |
| Devices | Otometrics Madsen Astera² clinical audiometer; Madsen Zodiac tympanometer |
| Reference construction | Duplicate masked transcription, adjudication against separate files, and independent audiologist curve classification |
| Development/evaluation structure | 50-record enriched image-development set; 101 patient-independent locked Codex image cases; matched post hoc DeepSeek visual arms in the same 101 patients; 60 report-refinement records; 91 patient-independent post-refinement report cases |
| Protected information | Direct identifiers remained in the secure duplicate audit and were not included in model-input or human-evaluator materials |
| Ethics approval | The University of Hong Kong Human Research Ethics Committee (EA260116) and the Ethics Committee of Ningbo Hospital of Integrated Traditional Chinese and Western Medicine (YJ260720192039) |
| External transmission | De-identified structured report inputs and, in the post hoc visual analysis, fully de-identified PTA and tympanometry images were sent through OpenRouter; zero-retention routing was not enforced for the visual analysis |
| Public availability | Raw records, images, and case-level context cannot be shared under hospital governance and ethics approval |
| Known limitations | Single site, two device models, short collection period, few N/NR entries, limited pediatric cases, and occasional historical fallback files |
| Prohibited interpretation | The dataset does not establish autonomous diagnostic safety, external validity, clinical effectiveness, or deployment readiness |

# **Model and Workflow Cards**

**Module 1 image workflows**

| **Field** | **Codex locked workflow** | **DeepSeek schema only** | **DeepSeek plus locked skill** |
| --- | --- | --- | --- |
| Intended task | PTA/tympanometry image transcription and derived audiology fields | Same | Same |
| Access/model | Isolated Codex agent task; Codex GPT5.5 | OpenRouter direct API; deepseek/deepseek-v4-flash-vision-exp | Same DeepSeek API model |
| Run date | July 7, 2026 | August 30, 2026 | August 30, 2026 |
| Prompt condition | Locked audiologist skill | Response schema without specialty rules | Same response schema plus locked skill |
| Settings | Isolated task; conventional sampling metadata unavailable | Temperature 0; reasoning disabled; JSON; maximum 6,000 tokens | Same; two truncated responses retried once at 12,000 tokens |
| Input | Two de-identified images per record | Same image pair | Same image pair |
| Withheld | Reference data, prior outputs, scores, adjudication files, and other skills | Same reference and scoring materials | Same reference and scoring materials |
| Governance limitation | Proprietary changing environment; snapshot and sampling unavailable | OpenRouter route; zero retention not enforced | Same |
| Intended interpretation | Descriptive agentic-workflow benchmark | Matched direct-API control for skill portability | Matched direct-API skill condition |

**Module 2 structured-input reporting workflows**

| **Field** | **Codex workflow** | **DeepSeek workflow** |
| --- | --- | --- |
| Intended task | Rule-derived fields plus Chinese professional and patient-facing reports | Same |
| Access/model | Isolated Codex agent task; Codex GPT5.5 | OpenRouter API; deepseek/deepseek-v4-flash, snapshot deepseek-v4-flash-20260423 |
| Run date/settings | July 13, 2026; xhigh; other sampling metadata unavailable | July 12, 2026; temperature 0; maximum 14,000 tokens |
| Input | Adjudicated structured data and de-identified context | Same structured packets |
| Withheld | Images, clinic label, precomputed derived answers, prior outputs, and scores | Images, clinic label, and precomputed derived answers |
| Retry rule | One stalled task retried | One empty first output retried |
| Human evaluation | Offline ratings by the same two audiologists who refined the prompt | Same |
| Interpretive limit | Different foundation model and agentic execution surface | Different foundation model and direct-API execution surface |

No workflow was intended for autonomous diagnosis, treatment, or unsupervised communication with patients. DeepSeek's official update and vision documentation reported the experimental visual model release on August 21, 2026 (https://api-docs.deepseek.com/updates/; https://api-docs.deepseek.com/guides/vision/; accessed August 30, 2026).

# **Supplementary Table S1. Recognition prompt development and prompt size**

| **Phase** | **Prompt condition** | **Description** | **Records/patients analyzed** | **System-prompt characters** | **Approximate first-task input tokens** |
| --- | --- | --- | --- | --- | --- |
| Early development | Compact skill | Reduced audiology rules used in early stress testing | 6 records | -- | -- |
| Early development | Detailed skill | Expanded rules used in early stress testing | 6 records | -- | -- |
| Development | Schema-only baseline | Output schema and safety constraints without audiology reading rules | 50 records | 443 | 1759 |
| Development | Standard audiologist skill | Audiology reading rules before final boundary refinements | 50 records | 4955 | 2887 |
| Validation | Locked audiologist skill | Final skill after development-set boundary refinements | 101 patients | 5772 | 3092 |

Early development failures were converted into explicit rules for the 5-dB grid, symbol/masking conventions, N/NR, sufficient bone-conduction evidence, hearing-loss type, professional versus printed tympanogram type, and cancelled 95-dB acoustic-reflex marks. Standard and detailed variants covered all prespecified edge rules; the standard version was selected because it achieved comparable rule coverage with lower prompt length.

# **Supplementary Table S2. Development comparison of schema-only and standard-skill prompting**

**Panel A. Performance in 50 development records**

| **Endpoint** | **Schema-only baseline** | **Standard audiologist skill** |
| --- | --- | --- |
| Numeric PTA thresholds within +/-5 dB | 896/897 (99.9%) | 895/897 (99.8%) |
| No-response status exact | 0/19 (0.0%) | 7/19 (36.8%) |
| PTA3 within +/-5 dB | 100/100 (100.0%) | 100/100 (100.0%) |
| Hearing-loss degree exact | 100/100 (100.0%) | 100/100 (100.0%) |
| Hearing-loss type exact | 85/100 (85.0%) | 94/100 (94.0%) |
| Clinically meaningful ABG exact | 92/100 (92.0%) | 99/100 (99.0%) |
| Instrument-printed tympanogram type exact | 100/100 (100.0%) | 100/100 (100.0%) |
| Professional curve-based tympanogram type exact | 94/100 (94.0%) | 92/100 (92.0%) |
| Acoustic-reflex screening exact; missing predictions incorrect | 261/371 (70.4%) | 365/371 (98.4%) |
| Tympanometry numeric mean absolute error | 0.000 | 0.000 |

**Panel B. Patient-clustered paired changes after adding the standard skill**

| **Endpoint** | **Skill minus schema, percentage points** | **Patient-cluster bootstrap 95% CI** | **Patient-cluster randomization p** |
| --- | --- | --- | --- |
| Numeric PTA thresholds within +/-5 dB | -0.1 | -0.3 to 0.0 | 1.0000 |
| No-response status exact | +36.8 | 0.0 to +52.0 | 0.5000 |
| PTA3 within +/-5 dB | 0.0 | 0.0 to 0.0 | -- |
| Hearing-loss degree exact | 0.0 | 0.0 to 0.0 | -- |
| Hearing-loss type exact | +9.0 | +4.0 to +16.0 | 0.0078 |
| Clinically meaningful ABG exact | +7.0 | +2.0 to +14.0 | 0.0625 |
| Instrument-printed tympanogram type exact | 0.0 | 0.0 to 0.0 | -- |
| Professional curve-based tympanogram type exact | -2.0 | -7.0 to +2.0 | 0.7500 |
| Acoustic-reflex screening exact | +28.0 | +17.7 to +39.0 | <0.0001 |

Blank reference cells were excluded. Missing predictions were incorrect. A no-response entry required an empty numeric value and a positive no-response flag. Confidence intervals used 20,000 bootstrap resamples of whole patients; randomization tests swapped all condition labels within a patient together. The N/NR and ABG changes were concentrated in two and four patients, respectively. All p values are exploratory and unadjusted.

# **Supplementary Table S3. Image recognition in 101 patient-independent cases**

**Panel A. Locked Codex GPT5.5 workflow: threshold-, ear-, and value-level performance**

| **Endpoint** | **Correct/total** | **Accuracy** | **Patient-cluster bootstrap 95% CI, where estimated** |
| --- | --- | --- | --- |
| Numeric PTA thresholds exact | 1809/1820 | 99.4% | 98.9-99.8% |
| Numeric PTA thresholds within +/-5 dB | 1810/1820 | 99.5% | 99.0-99.8% |
| Numeric PTA thresholds within +/-10 dB | 1811/1820 | 99.5% | 99.1-99.8% |
| No-response status exact | 9/15 | 60.0% | Not estimated; 15 entries occurred in 3 patients |
| PTA3 within +/-5 dB | 202/202 | 100.0% | 100.0-100.0% |
| Hearing-loss degree exact | 202/202 | 100.0% | 100.0-100.0% |
| Hearing-loss type exact | 201/202 | 99.5% | 98.5-100.0% |
| Clinically meaningful ABG exact | 183/202 | 90.6% | 84.7-96.0% |
| Instrument-printed tympanogram type exact | 202/202 | 100.0% | 100.0-100.0% |
| Professional curve-based tympanogram type exact | 195/202 | 96.5% | 93.6-99.0% |
| Comparable acoustic-reflex cells exact | 717/720 | 99.6% | 99.0-100.0% |
| Comparable tympanometry numeric values exact | 578/578 | 100.0% | 100.0-100.0% |

**Panel B. Locked Codex GPT5.5 workflow: patient-level performance**

| **Endpoint** | **Correct patients/total** | **Accuracy** | **Wilson 95% CI** |
| --- | --- | --- | --- |
| All numeric and no-response criteria correct | 91/101 | 90.1% | 82.7-94.5% |
| Both-ear PTA3 within +/-5 dB | 101/101 | 100.0% | 96.3-100.0% |
| Both-ear hearing-loss degree exact | 101/101 | 100.0% | 96.3-100.0% |
| Both-ear hearing-loss type exact | 100/101 | 99.0% | 94.6-99.8% |
| Both-ear clinically meaningful ABG exact | 91/101 | 90.1% | 82.7-94.5% |
| Both-ear printed tympanogram type exact | 101/101 | 100.0% | 96.3-100.0% |
| Both-ear professional curve type exact | 95/101 | 94.1% | 87.6-97.2% |
| All comparable acoustic-reflex cells exact | 91/94 | 96.8% | 91.0-98.9% |
| All comparable tympanometry numeric values exact | 101/101 | 100.0% | 96.3-100.0% |

Eighteen of 19 strict ABG mismatches were model no versus reference indeterminate because fewer than two comparable bone-conduction frequencies were available. The remaining yes/no disagreement involved a lower-bound N/NR surrogate and also changed hearing-loss type.

**Panel C. Post hoc matched DeepSeek direct-API arms**

| **Endpoint** | **Schema only** | **Locked audiologist skill** | **Skill minus schema, percentage points (95% patient-cluster bootstrap CI)** |
| --- | --- | --- | --- |
| Numeric PTA thresholds exact | 1274/1820 (70.0%) | 1312/1820 (72.1%) | +2.1 (-0.8 to +5.2) |
| Numeric PTA thresholds within +/-5 dB | 1594/1820 (87.6%) | 1642/1820 (90.2%) | +2.6 (+0.2 to +5.7) |
| Air-conduction numeric thresholds exact | 989/1205 (82.1%) | 1016/1205 (84.3%) | +2.2 (-0.6 to +5.3) |
| Bone-conduction numeric thresholds exact | 285/615 (46.3%) | 296/615 (48.1%) | +1.8 (-2.9 to +6.8) |
| No-response status exact | 0/15 (0.0%) | 0/15 (0.0%) | 0.0 (not estimated; 3 patients) |
| AC PTA3 exact | 113/202 (55.9%) | 133/202 (65.8%) | +9.9 (+3.0 to +16.8) |
| AC PTA3 within +/-5 dB | 194/202 (96.0%) | 199/202 (98.5%) | +2.5 (+0.5 to +5.0) |
| Hearing-loss degree exact | 167/202 (82.7%) | 189/202 (93.6%) | +10.9 (+4.5 to +17.8) |
| Hearing-loss type exact | 76/202 (37.6%) | 142/202 (70.3%) | +32.7 (+23.3 to +42.1) |
| Clinically meaningful ABG exact | 118/202 (58.4%) | 113/202 (55.9%) | -2.5 (-12.4 to +7.4) |
| Instrument-printed tympanogram type exact | 202/202 (100.0%) | 202/202 (100.0%) | 0.0 (0.0 to 0.0) |
| Professional curve-based tympanogram type exact | 194/202 (96.0%) | 194/202 (96.0%) | 0.0 (0.0 to 0.0) |
| Tympanometry numeric values exact | 578/578 (100.0%) | 578/578 (100.0%) | 0.0 (0.0 to 0.0) |
| Acoustic-reflex screening exact | 495/720 (68.8%) | 497/720 (69.0%) | +0.3 (0.0 to +0.7) |

This post hoc cross-model analysis tested portability of the locked skill within one direct-API visual model. Confidence intervals used 20,000 bootstrap resamples of whole patients and were not adjusted for multiplicity; no interval was estimated for the three-patient N/NR endpoint. It reused the Codex cases, reference standard, and locked skill and therefore was not an independent-dataset validation. Codex and DeepSeek absolute performance should not be interpreted as a matched base-model comparison because the Codex workflow was agentic and its conventional sampling controls were unavailable.

# **Supplementary Table S4. Structured-input rule conformance in 91 post-refinement patients**

| **Computed endpoint** | **Codex GPT5.5** | **DeepSeek v4 Flash** |
| --- | --- | --- |
| Patients with all 25 fields correct | 91/91 (100.0%) | 87/91 (95.6%) |
| All prespecified computed fields | 2275/2275 (100.0%) | 2256/2275 (99.2%) |
| AC PTA3 | 182/182 (100.0%) | 182/182 (100.0%) |
| AC PTA4 | 182/182 (100.0%) | 182/182 (100.0%) |
| Clinically meaningful ABG | 182/182 (100.0%) | 177/182 (97.3%) |
| Hearing-loss degree by AC PTA3 | 182/182 (100.0%) | 181/182 (99.5%) |
| Hearing-loss type by PTA/ABG | 182/182 (100.0%) | 178/182 (97.8%) |
| Tympanogram pattern mapping | 182/182 (100.0%) | 182/182 (100.0%) |
| PTA-tympanometry cross-check | 182/182 (100.0%) | 177/182 (97.3%) |
| Conductive-component laterality | 91/91 (100.0%) | 89/91 (97.8%) |
| Any conductive component | 91/91 (100.0%) | 89/91 (97.8%) |

The four patient-level all-field discordances occurred in the DeepSeek workflow; this pattern is reported descriptively because the workflows were unmatched.

Both workflows received adjudicated raw thresholds, N/NR flags, tympanometry measurements, instrument-printed and professional curve types, acoustic-reflex entries, and de-identified clinical context. They did not receive images, precomputed PTA/ABG/degree/type/laterality/cross-check answers, or the clinic diagnosis. References were generated from the same prespecified rules given to both workflows; this is a rule-conformance benchmark, not independent diagnostic validation.

# **Supplementary Table S5. Offline audiologist ratings and descriptive paired patterns in 91 patients**

**Panel A. Patient-level ratings**

| **Workflow** | **Report** | **Endpoint** | **Patients** | **Percentage** | **Wilson 95% CI** |
| --- | --- | --- | --- | --- | --- |
| Codex GPT5.5 | Professional | Both audiologists rating 1 or 2 | 91/91 | 100.0% | 95.9-100.0% |
| DeepSeek v4 Flash | Professional | Both audiologists rating 1 or 2 | 91/91 | 100.0% | 95.9-100.0% |
| Codex GPT5.5 | Patient-facing | Both audiologists rating 1 or 2 | 91/91 | 100.0% | 95.9-100.0% |
| DeepSeek v4 Flash | Patient-facing | Both audiologists rating 1 or 2 | 91/91 | 100.0% | 95.9-100.0% |
| Codex GPT5.5 | Professional | Both audiologists rating 1 | 75/91 | 82.4% | 73.3-88.9% |
| DeepSeek v4 Flash | Professional | Both audiologists rating 1 | 87/91 | 95.6% | 89.2-98.3% |
| Codex GPT5.5 | Patient-facing | Both audiologists rating 1 | 88/91 | 96.7% | 90.8-98.9% |
| DeepSeek v4 Flash | Patient-facing | Both audiologists rating 1 | 88/91 | 96.7% | 90.8-98.9% |
| Either workflow | Either report | Any rating 3 or 4 | 0/364 workflow-report-patient combinations | 0.0% | -- |

**Panel B. Descriptive paired patient-level patterns**

| **Report/endpoint** | **Codex higher** | **DeepSeek higher** | **Ties** |
| --- | --- | --- | --- |
| Professional, both rating 1 or 2 | 0 | 0 | 91 |
| Professional, both rating 1 | 3 | 15 | 73 |
| Patient-facing, both rating 1 or 2 | 0 | 0 | 91 |
| Patient-facing, both rating 1 | 1 | 1 | 89 |

These offline ratings used adjudicated structured inputs and do not establish prospective safety. The paired counts are descriptive and do not identify a superior foundation model because model and execution architecture differed. With 0/91 unfavorable patients, the complementary upper 95% bound is approximately 4.1%.

# **Supplementary Table S6. Inter-rater agreement**

**Panel A. Four-level expert accuracy ratings**

| **Workflow/report** | **Patients** | **Observed agreement** | **Unweighted kappa** | **Quadratic-weighted kappa** |
| --- | --- | --- | --- | --- |
| Codex GPT5.5, professional | 91 | 0.912 | 0.622 | 0.622 |
| Codex GPT5.5, patient-facing | 91 | 0.967 | 0.000 | 0.000 |
| DeepSeek v4 Flash, professional | 91 | 0.956 | -0.017 | -0.017 |
| DeepSeek v4 Flash, patient-facing | 91 | 0.967 | -0.015 | -0.015 |

**Panel B. Preference agreement**

| **Rater group** | **Dimension** | **Patients** | **Observed agreement** | **Kappa** |
| --- | --- | --- | --- | --- |
| Experts | Professional overall | 48 | 0.479 | 0.100 |
| Experts | Professional accuracy | 48 | 0.438 | 0.146 |
| Experts | Professional next steps | 48 | 0.438 | -0.013 |
| Experts | Professional clarity | 48 | 0.417 | 0.021 |
| Experts | Patient-facing overall | 48 | 0.708 | 0.246 |
| Experts | Patient-facing accuracy | 48 | 0.313 | 0.017 |
| Experts | Patient-facing understandability | 48 | 0.500 | 0.055 |
| Experts | Patient-facing next steps | 48 | 0.521 | 0.182 |
| Laypersons | Overall preference | 48 | 0.410 | -0.147 |
| Laypersons | Easier to understand | 48 | 0.398 | -0.158 |
| Laypersons | Better explanation | 48 | 0.521 | -0.119 |
| Laypersons | Clearer next steps | 48 | 0.406 | -0.008 |

Kappa is sensitive to prevalence and marginal imbalance. Near-uniform expert ratings and heterogeneous lay preferences can produce low kappa despite higher observed agreement; both measures are reported descriptively.

# **Supplementary Table S7. Patient-independent preferences and text-complexity proxies**

**Panel A. Preferences in 48 patients**

| **Rater group** | **Dimension** | **Codex patients** | **DeepSeek patients** | **Neutral/no consensus** | **Exact p among decisive patients** |
| --- | --- | --- | --- | --- | --- |
| Expert | Professional overall | 4 | 17 | 27 | 0.007 |
| Expert | Professional accuracy | 5 | 0 | 43 | 0.063 |
| Expert | Professional clarity | 3 | 15 | 30 | 0.008 |
| Expert | Professional next steps | 3 | 18 | 27 | 0.001 |
| Expert | Patient-facing overall | 2 | 31 | 15 | <0.001 |
| Expert | Patient-facing accuracy | 1 | 3 | 44 | 0.625 |
| Expert | Patient-facing understandability | 1 | 22 | 25 | <0.001 |
| Expert | Patient-facing next steps | 1 | 16 | 31 | <0.001 |
| Layperson | Overall preference | 20 | 27 | 1 | 0.382 |
| Layperson | Easier to understand | 24 | 22 | 2 | 0.883 |
| Layperson | Better explanation of hearing condition | 1 | 44 | 3 | <0.001 |
| Layperson | Clearer next steps | 1 | 30 | 17 | <0.001 |

Expert preference required agreement by both experts; layperson results used the five-rater patient-level majority. Tests are exploratory and unadjusted.

**Panel B. Paired text-complexity proxies, median (IQR)**

| **Report/measure** | **Codex GPT5.5** | **DeepSeek v4 Flash** | **Paired p value** |
| --- | --- | --- | --- |
| Patient-facing characters | 235 (220-255) | 392 (348-452) | <0.001 |
| Patient-facing Chinese characters/sentence | 19.4 (18.4-20.2) | 28.9 (26.3-30.6) | <0.001 |
| Patient-facing medical terms/100 Chinese characters | 2.46 (1.91-3.11) | 2.90 (2.32-3.51) | 0.048 |
| Patient-facing readability-burden proxy | 2.00 (1.86-2.16) | 3.01 (2.81-3.17) | <0.001 |
| Professional characters | 842 (754-902) | 865 (747-1000) | 0.451 |
| Professional readability-burden proxy | 5.99 (5.77-6.36) | 6.10 (5.40-6.45) | 0.583 |

The readability-burden proxy combines text length, sentence length, medical-term density, acronym count, and numeric-token count. It is transparent but not a validated Chinese education-grade measure.

# **Supplementary Table S8. Partition characteristics and sensitivity analyses**

**Panel A. Selected partition characteristics**

| **Characteristic** | **Recognition development 50** | **Validation 101** | **Expert feedback 60** | **Formal evaluation 91** |
| --- | --- | --- | --- | --- |
| Age, mean (SD), years | 40.1 (17.4) | 41.4 (18.3) | 38.8 (15.6) | 42.3 (19.3) |
| Female | 33/50 (66.0%) | 64/101 (63.4%) | 40/60 (66.7%) | 57/91 (62.6%) |
| Age younger than 18 years | 2/50 (4.0%) | 11/101 (10.9%) | 3/60 (5.0%) | 10/91 (11.0%) |
| Any clinically meaningful conductive component | 13/50 (26.0%) | 20/101 (19.8%) | 16/60 (26.7%) | 17/91 (18.7%) |
| Any abnormal professional tympanogram | 34/50 (68.0%) | 64/101 (63.4%) | 37/60 (61.7%) | 61/91 (67.0%) |
| Any N/NR threshold | 5/50 (10.0%) | 3/101 (3.0%) | 4/60 (6.7%) | 4/91 (4.4%) |

Largest absolute standardized differences were 0.288 for N/NR and 0.265 for age under 18 years in the recognition split, and 0.222 for age under 18 years in the reporting split.

**Panel B. Post-refinement sensitivity analyses**

| **Restriction/endpoint** | **Codex GPT5.5** | **DeepSeek v4 Flash** |
| --- | --- | --- |
| Exclude any historical fallback test (n=86): all 25 fields correct | 86/86 (100.0%) | 82/86 (95.3%) |
| Exclude fallback tests: professional reports rated 1 by both | 71/86 (82.6%) | 82/86 (95.3%) |
| Exclude fallback tests: patient-facing reports rated 1 by both | 83/86 (96.5%) | 83/86 (96.5%) |
| Adults only (n=81): all 25 fields correct | 81/81 (100.0%) | 77/81 (95.1%) |
| Adults only: professional reports rated 1 by both | 65/81 (80.2%) | 77/81 (95.1%) |
| Adults only: patient-facing reports rated 1 by both | 78/81 (96.3%) | 78/81 (96.3%) |

Both audiologists assigned ratings 1 or 2 to every professional and patient-facing report under both restrictions.

# **Supplementary Table S9. Human reference standard and rater characteristics**

**Panel A. Pre-adjudication agreement in 151 patients**

| **Reference field** | **Exact agreement/compared nonblank pairs** | **Agreement** |
| --- | --- | --- |
| PTA air-conduction threshold | 1791/1812 | 98.8% |
| PTA bone-conduction threshold | 909/939 | 96.8% |
| Instrument-printed tympanogram type | 297/302 | 98.3% |
| Tympanometric peak pressure | 279/280 | 99.6% |
| Static admittance/compliance | 277/280 | 98.9% |
| Ear-canal volume | 299/302 | 99.0% |
| Ipsilateral acoustic-reflex entry | 1090/1091 | 99.9% |
| Professional curve type among dual-rated ears | 296/300 | 98.7% |

Both audiologists classified 300/302 ears; Cohen kappa for A/As/Ad/B/C was 0.979. Six ears required adjudication: four disagreements and two ears with one initial classification. Professional curve classification was an expert-consensus, non-rule-based reference standard: the audiologists integrated morphology, peak location, static admittance/compliance, and ear-canal volume without universal numerical cutoffs, and jointly reviewed borderline or discordant curves to reach consensus. Contralateral reflex fields were blank throughout.

**Panel B. Rater characteristics**

| **Rater group** | **Number** | **Sex** | **Age, years** | **Education/experience** |
| --- | --- | --- | --- | --- |
| Clinical audiologists | 2 | Both female | 36, 39 | Bachelor's/master's degrees; 13 and 16 years of clinical experience |
| Laypersons | 5 | 3 female, 2 male | 33, 37, 40, 64, 66 | Junior-secondary school to bachelor's degree |

Lay raters were recruited through open poster advertisements. None had formal audiology education or training, and none received compensation. Hearing status and health literacy were not collected.

# **Supplementary Table S10. Operational rules and reproducibility audit**

**Panel A. Prespecified input and derived-label rules**

| **Derived label** | **Prespecified study rule** |
| --- | --- |
| Observed N/NR | Empty numeric threshold plus a positive no-response flag; N and NR both denoted no response, not not tested |
| N/NR in lower-bound calculations | For PTA3/PTA4, degree, type, and ABG only, frequency-specific maximum output plus 5 dB; the observed threshold remained nonnumeric |
| Local maximum outputs, dB HL | AC: 250=105, 500=120, 1000=120, 2000=115, 4000=120, 8000=105 Hz; BC: 250=50, 500=70, 1000=80, 2000=80, 4000=75 Hz |
| Comparable AC/BC frequency | Same ear and nominal frequency with unambiguous ear and masking assignment; measured response or clear N/NR lower bound; blank, crossed-out, superseded, or uncertain marks excluded |
| Normal or near-normal | AC PTA3 <=25 dB HL and no clinically meaningful ABG |
| Normal or near-normal with conductive component | AC PTA3 <=25 dB HL and clinically meaningful ABG=yes |
| Conductive hearing loss | AC PTA3 >25 dB HL, ABG=yes, and BC PTA3 <=25 dB HL |
| Mixed hearing loss | AC PTA3 >25 dB HL, ABG=yes, and BC PTA3 >25 dB HL |
| Sensorineural hearing loss | AC PTA3 >25 dB HL, ABG=no, and BC PTA3 >25 dB HL |
| Indeterminate type | Required AC/BC evidence missing or remaining combination not assigned above |
| Clinically meaningful ABG=yes | At least two comparable AC/BC frequencies and either at least two ABGs >=15 dB or mean ABG >=15 dB |
| Clinically meaningful ABG=indeterminate | Fewer than two comparable AC/BC frequencies |
| ABG with middle-ear support | ABG=yes and professional curve is As, Ad, B, or C |
| ABG without tympanometric support | ABG=yes and professional curve is A |
| Tympanogram abnormal without significant ABG | ABG=no and professional curve is As, Ad, B, or C |
| No middle-ear pattern | ABG=no and professional curve is A |

**Panel B. Codex GPT5.5 session-level audit of the 105-record recognition run**

| **Audit criterion** | **Result** |
| --- | --- |
| Locked-manifest records | 105 |
| Matching archived record sessions | 105/105 |
| Unique completed outputs | 105/105 |
| Sessions viewing both permitted source images | 105/105 |
| Sessions using an unexpected tool | 0/105 |
| Sessions with a logged reference/adjudication/scoring/evaluation path | 0/105 |
| Sessions additionally viewing a same-record temporary crop | 4/105 |

The patient-independent statistical analysis retained 101 of these outputs. Archived session logs and SHA-256 hashes verified logged tool-call compliance but were not equivalent to a filesystem sandbox. Exact non-identifiable prompt files and their integrity manifest are available in GitHub release v1.0.0 at https://github.com/sqgwang/audiologist-skills/releases/tag/v1.0.0. AC indicates air conduction; BC, bone conduction; PTA3, mean threshold at 500, 1000, and 2000 Hz; ABG, air-bone gap. These operational rules define the benchmark and are not a universal diagnostic taxonomy.
